# Physical activity, social support and BMI among middle school students in China

**DOI:** 10.3389/fpsyg.2026.1779679

**Published:** 2026-04-14

**Authors:** Sixiang Tao, Han Liu, Nur Shakila Mazalan, Feng Hong, Denise Koh

**Affiliations:** 1 Faculty of Sport Education, Huainan Normal University, Huainan, Anhui, China; 2 Faculty of Education, National University of Malaysia, Bangi, Selango, Malaysia; 3 Institute of Chinese Sports Development, Beijing Sport University, Beijing, China; 4 Department of Sports Operation and Management, Jinhua University of Vocational Technology, Jinhua, Zhejiang, China

**Keywords:** social support, physical activity, BMI Z-score, teacher support, Chinese adolescents

## Abstract

**Introduction:**

Adolescent obesity is a growing public health concern in China, driven largely by insufficient physical activity and increasing sedentary behaviors. Evidence on how multiple sources of social support relate to adolescents' physical activity and weight status remains relatively limited within the Chinese educational and cultural context. This study aims to examine the associations between multi-source social support, physical activity, and BMI Z-scores, and to explore gender-specific patterns among Chinese junior high school students.

**Methods:**

A cross-sectional survey was conducted among 518 students aged 12–15 years from three junior high schools in Kunming, Yunnan Province. Participants completed the Physical Activity Rating Scale (PARS-3) and the Child and Adolescent Social Support Scale (CASSS), which assesses perceived support from five sources (parents, teachers, classmates, close friends, and school). BMI data were obtained from recent school health assessments. Hierarchical regression and restricted cubic spline analyses were employed to explore the associations and non-linear dose–response patterns between physical activity, multi-source social support, and BMI Z-scores.

**Results:**

Physical activity showed the strongest negative association with BMI Z-scores (β = −0.34, *p* < 0.001), while teacher support remained independently associated (β = −0.10, *p* < 0.05). Parental, classmate, and close-friend support were not significant. Non-linear analyses revealed gender-specific trends: boys displayed a linear dose–response pattern, whereas girls showed a U-shaped curve with optimal BMI at moderate activity levels. The association between teacher support and BMI Z-scores was more evident among girls. The final model explained 14.1% of BMI Z-score variance.

**Discussion:**

Physical activity showed the strongest association with BMI Z-scores among adolescents, and teacher support was uniquely associated with weight status in the Chinese school context. Gender-specific non-linear patterns highlight the need for culturally grounded, school-centered, and gender-sensitive strategies to promote healthy weight in early adolescence.

## 1 Introduction

Adolescents' obesity has become an important public health problem globally, and this trend has been particularly evident in China. The epidemiologic survey showed that the combined prevalence of overweight and obesity among Chinese adolescents reached approximately 27–30% in 2016, with obesity prevalence being highest among adolescent males at 15.8% (Li et al., 2024). Alarmingly, this trend continues to deteriorate, with the prevalence of overweight and obesity among children and adolescents rising sharply from 15.5% in 2010 to 29.4% in 2022 (Zhu and Yin, 2023). Data from the National Student Physical Fitness and Health Study showed that the prevalence of obesity in children and adolescents aged 7–18 years increased from 0.1% to 9.6% between 1985 and 2019 (Yuan et al., 2024). Overweight and obesity not only affect the physical health of adolescents and increase the risk of chronic diseases such as cardiovascular disease and type 2 diabetes, but may also lead to psychosocial problems, and these effects often continue into adulthood (Salama et al., 2023; Lister et al., 2023).

Physical activity is widely recognized as a key behavioral factor associated with weight management and obesity prevention. Numerous cross-sectional and longitudinal studies have confirmed a stable negative correlation between physical activity levels and body mass index (BMI) in children and adolescents (Rauner et al., 2013). Regular physical activity increases total energy expenditure and is an important component of weight management programs (Wyszyńska et al., 2020). Studies on Chinese children and adolescents have shown that normal-weight children have significantly better physical performance than their overweight and obese peers (Wang et al., 2022; Chen et al., 2022). However, the physical activity levels of Chinese children and adolescents are of concern. National monitoring data show that due to rapid economic development and the popularization of electronic devices, Chinese adolescents have a declining trend in physical activity and a significant increase in sedentary behaviors (Shen et al., 2020; Guo et al., 2024). This current situation of physical inactivity is reflected across regions, genders, and age groups, and has become an important constraint on the healthy development of adolescents.

Social support refers to the resources embedded in social relationships that facilitate health-related behaviors through emotional reassurance, practical assistance, and guidance. In youth physical activity research, social support is commonly conceptualized along two dimensions: functional types and sources. Functional types typically include informational support (e.g., advice or guidance), instrumental support (e.g., tangible help or resources), and companionship support (e.g., co-participation and encouragement), whereas sources refer to the social agents providing support, such as parents, teachers, and peers (Duncan et al., 2005). Although different types of social support may operate through distinct behavioral mechanisms, the present study focuses on sources of social support within the school-age context and does not distinguish between specific functional types.

Accumulating evidence suggests that parental and peer support are more consistently associated with adolescents' physical activity behaviors, whereas the relationship between social support and BMI or weight status appears weaker and more context-dependent, varying by support source and sociocultural setting (Vander Wal, 2012; Lebacq et al., 2019; Chen et al., 2023). Moreover, social support can be characterized in terms of both frequency (how often support is received) and perceived importance (how meaningful the support is). While both dimensions are captured by commonly used instruments, including the Child and Adolescent Social Support Scale, the present study focuses on frequency-based indicators as a measure of received support, acknowledging that qualitative aspects of support may exert indirect or context-specific effects that are not examined in the current analyses.

This gap may be particularly relevant in the Chinese educational context, where under China's compulsory education system, junior high school students receive full-day schooling within a highly structured learning environment. Teachers therefore represent one of the most consistent adult figures in adolescents' daily lives, not only shaping academic engagement but also directly influencing physical education participation, health-related norms, and behavioral expectations (Wu X. et al., 2024). Rooted in Confucian cultural traditions that emphasize respect for authority and teacher–student relationships, teacher support may exert a stronger influence on adolescents' health-related behaviors than parental support during early adolescence (Wang et al., 2024). In the Chinese cultural context, different sources of social support—such as family, teachers, and peers—may operate through distinct pathways and exert differential effects on adolescents' physical activity and weight status. However, empirical evidence examining these source-specific influences among Chinese middle school students remains scarce. Moreover, much of the existing literature has focused on Western populations, underscoring the need for culturally grounded research that systematically examines the relationships among multi-source social support, physical activity, and BMI in Chinese adolescents (Chen et al., 2020; Hu et al., 2024).

Based on the above background, the study aimed to explore the role of multiple sources of social support (parents, teachers, classmates, close friends, and school) in the relationship between physical activity and BMI among Chinese junior high school students. Specific objectives included: 1. analyzing the patterns of association between different sources of social support and junior high school students‘ BMI Z-scores; 2. exploring the relationship between physical activity and BMI Z-scores; 3. examining the relative contributions of multiple sources of social support and physical activity to BMI Z-scores; 4. analyzing possible gender differences in the above relationships. Based on social-ecological theory and existing evidence, we hypothesize that physical activity is negatively associated with BMI Z-scores, and that different sources of social support may affect adolescents' weight status to different degrees.

## 2 Methods

### 2.1 Study design and participants

This cross-sectional study recruited 518 junior high school students from three public schools in Kunming, Yunnan Province. A two-stage cluster sampling strategy was applied. At the first stage, three public junior high schools were purposively selected to represent urban, peri-urban, and rural settings within Kunming, with the urban school located in an urban district, the peri-urban school in a county-level town, and the rural school in a township within an outer district of Kunming. At the second stage, classes within each selected school were randomly sampled, and all eligible students within the selected clusters were invited to participate. This approach ensured random selection at the student level while enhancing contextual diversity across school settings.

Although socioeconomic conditions may differ across these contexts, all participating schools were public junior high schools within China's compulsory education system, where no tuition fees are charged and student enrollment is based on residential catchment areas rather than family income or fee-paying status, thereby minimizing school-level socioeconomic selection. All participating schools followed the same public education system and standardized curriculum, which reduced the likelihood of substantial between-school variation in physical activity, BMI, and social support measures. Students in grades 7 to 9 (aged 12–15 years) were selected using a random cluster sampling method. This age range represents a critical developmental stage in which adolescents experience rapid changes in social relationships, increasing peer influence, and heightened sensitivity to school-based interactions—factors that may be associated with physical activity participation and weight-related behaviors (Orben et al., 2020; Voelker et al., 2015).

A total of 600 questionnaires were distributed. With assistance from teachers, students independently completed the survey, which included the Physical Activity Rating Scale (PARS-3) and the Child and Adolescent Social Support Scale (CASSS). The completion time was approximately 20–25 min. As a self-reported measure, PARS-3 may be subject to social desirability bias, particularly among adolescents, who may overestimate their physical activity levels. Of the 582 returned questionnaires, 46 were excluded due to incomplete or invalid responses, defined by predefined data completeness and quality-control criteria (e.g., substantial missing item responses, patterned or contradictory answers, or failure to complete key scale items). An additional 18 questionnaires were excluded because corresponding height or weight data were unavailable from the Chinese National Student Physical Fitness Test records. The final valid sample consisted of 518 participants, exceeding the recommended minimum sample size of 300 for robust behavioral statistical analysis (Tabachnick and Fidell, 1996).

This study received approval from the Research Ethics Committee of Huainan Normal University (Approval No. 2024-047). Written informed consent was obtained from all students and their parents or legal guardians prior to data collection.

### 2.2 Physical activity

Physical activity was measured using the Chinese version of the Physical Activity Rating Scale (PARS-3). The scale consists of 3 items assessing intensity, duration, and frequency of physical activity. The intensity and frequency entries are scored on a 5 point scale (1–5), and the time entry is scored on a 5-point scale (0–4). The scale asked subjects to respond to the question, “In the last week, how physically active have you been?” The intensity entry asked, “What intensity do you usually reach when you participate in physical activity?” The intensity entry asks “What intensity do you usually reach when you are physically active?”, with answers ranging from 1 (“light activity, such as walking”) to 5 (“vigorous activity, such as running fast”), and the time entry asks “How long do you usually spend participating in each physical activity?”, with answers ranging from 0 points (“hardly ever exercise”) to 4 points (“more than 60 min.”); the frequency entry asks “How many times per week do you participate in physical activity?” The frequency entry asks, “How many times per week do you participate in physical activity?,” with answers ranging from 1 point (“once”) to 5 points (“every day”). The formula for calculating the total physical activity score is: physical activity = intensity × time × frequency, and the total score ranges from 0 to 100. The scale showed good reliability and validity in the Chinese student population, with Cronbach 's alpha ranging from 0.838 to 0.942 (Liang, 1994; Wu H. et al., 2024).

### 2.3 Social support

Social support was measured using the Chinese version of the Child and Adolescent Social Support Scale (CASSS). The CASSS is a multidimensional scale that measures children's and adolescents' perceived social support from five different sources: parental support, teacher support, classmates' support and close friends' support, and school support. The scale consists of 60 items, 12 for each source of support. The scale asks students to rate statements such as “My parents help me make decisions” and “Teachers care and support me”. Students were asked to rate each entry on two dimensions: frequency and importance. Frequency ratings were based on a 6-point Likert scale ranging from 1 (“never”) to 6 (“always”); importance ratings were based on a 3-point Likert scale ranging from 1 (“not important”) to 3 (“very important”). Analyses focused primarily on frequency scores, as they reflect the extent of received support and have been more consistently linked to behavioral outcomes in previous studies. Although the CASSS also assesses perceived importance of support as an indicator of support quality, importance ratings were not included in the present analyses to maintain conceptual consistency with the study aims and analytic focus on received support. Subscale scores were calculated by summing the frequency ratings of the corresponding 12 items. Analyses focused on support from individual sources rather than an overall social support score, to capture potential source-specific associations (Malecki et al., 1999).

The scale has good reliability and validity, with confirmatory factor analysis supporting its five-factor structure (parents, teachers, classmates and close friends, and school subscales) and showing good construct validity. The scale has been widely used to assess perceived social support in children and adolescents in grades 3–12 (Luo et al., 2017).

### 2.4 BMI and BMI Z-score

Body mass index (BMI) was obtained by calculating height and weight data from the March 2025 Chinese Student Physical Fitness Standard (CNSPFS) (Zhang et al., 2021) monitoring program in the 3 sampled schools. Measurements were conducted by trained school enumerators following standardized CNSPFS protocols, who had received formal training provided by local education authorities as part of the routine CNSPFS implementation and were conducted 1 month prior to the questionnaire. Since the short time interval would not significantly affect the study results, children were not given repeated height and weight measurements on the day of the questionnaire survey, and the most recent CNSPFS monitoring data were used instead.

According to CNSPFS standards, height was measured using a wall-mounted stadiometer accurate to 0.1 cm, weight was measured using a calibrated electronic scale accurate to 0.1 kg, and participants wore light clothing and no shoes during the measurements. These data were provided by the physical education teachers responsible for the CNSPFS test in the participating schools, in accordance with the formal research collaboration protocol and ethical approval requirements.

BMI was calculated as weight in kilograms divided by the square of height in meters (kg/ m^2^). BMI values were converted to BMI Z-values for standardized assessment of weight status in the study population using the WHO Anthro Survey Analyzer based on the participants' year of birth, sex, and height and weight data at the time of measurement (World Health Organization, 2011).

### 2.5 Statistical analyses

All statistical analyses were performed using *R* studio (2025.05.0 + 496). Data were first tested for normality and outliers were identified. Outlier screening was conducted after excluding cases with missing height or weight data; identified outliers were retained in the analyses without winsorization or transformation, as they represented plausible values rather than data entry errors. Continuous variables were expressed as mean ± standard deviation (*M* ± SD) and categorical variables were expressed as frequency and percentage (*n*, %). Second, descriptive statistical analyses were used to summarize the baseline characteristics of the study participants, including age, gender, height, weight, BMI, BMI Z-value, and the distribution of each social support variable. Prior to regression analyses, Pearson correlation analyses were conducted to describe the bivariate associations among physical activity, multi-source social support variables, and BMI Z-scores.

Hierarchical regression analysis was used to examine the independent associations of physical activity and social support with BMI Z-scores. Four hierarchical models were developed using the function: model 1 contained control variables (age, gender); model 2 added the physical activity variable (PARS-3 score) to model 1; model 3 added the frequency of teacher support to model 2; and model 4 added the frequency of parental support to model 3. Standardized regression coefficients (β), coefficients of determination (*R*^2^), adjusted coefficients of determination (Adjusted *R*^2^), the amount of change in *R*^2^ (Δ*R*^2^), and the results of the F change test are reported. Although mixed-effects models can account for clustering at the school level, hierarchical regression was chosen because the number of clusters was small (three schools) and no substantial between-school variability was expected, conditions under which random-effects estimates may be unstable. To further explore potential non-linear dose–response relationships, restricted cubic spline (RCS) analyses were performed to explore potential non-linear dose–response patterns in these associations. Spline curves were fitted using generalized additive models and visualized to illustrate gender-specific non-linear patterns.

## 3 Result

### 3.1 Descriptive analysis

A total of 518 junior high school students were included in this study and their baseline characteristics are shown in Table 1. The mean age of the participants was 13.66 ± 0.86 years, which is in the typical age range for junior high school. In terms of gender distribution, 57.9% (*n* = 300) were boys and 42.1% (*n* = 218) were girls.

**Table 1:** Baseline characteristics statistics of study participants (*N* = 518).

Variables	Total (*n* = 518)	Boys (*n* = 300)	Girls (*n* = 218)
Age (years)	13.66 ± 0.86	13.71 ± 0.88	13.58 ± 0.83
Height (cm)	159.87 ± 7.92	161.11 ± 7.00	158.16 ± 8.77
Weight (kg)	51.64 ± 9.60	52.21 ± 9.07	50.86 ± 10.25
BMI (kg/m^2^)	18.95 ± 2.76	18.89 ± 2.72	19.04 ± 2.83
BMI Z-score	0.30 ± 0.86	0.37 ± 0.84	0.22 ± 0.87

In terms of physical measurements, the mean height of the participants was 159.87 ± 7.92 cm, the mean weight was 51.64 ± 9.60 kg, and the mean BMI was 18.95 ± 2.76 kg/m^2^. The mean BMI Z-score was 0.30 ± 0.86, indicating that, on average, the weight status of the study population was close to the WHO standardized reference values. This distribution suggests that most participants fell within the normal BMI Z-score range, while still exhibiting sufficient variability to examine associations between BMI, physical activity, and social support. Overall, the age and physical characteristics of the sample were consistent with those of Chinese junior high school students, supporting the suitability of the study population for investigating relationships among multisource social support, physical activity, and BMI. Table 2 shows the bivariate correlations among BMI Z-scores, physical activity, and different sources of social support. BMI Z-scores were negatively correlated with physical activity and weakly correlated with parental and teacher support, whereas correlations with classmate, close friend, and school support were not significant. Intercorrelations among social support sources were moderate to strong, indicating overlapping support environments. These correlations provide an overview of the relationships among the study variables and contextualize the interpretation of the subsequent hierarchical regression analyses.

**Table 2:** Pearson correlations among BMI Z-score, physical activity, and social support variables (*N* = 518).

Variables	1	2	3	4	5	6	7
1. BMI Z-score	1						
2. Physical activity (PARS-3)	−0.20^***^	1					
3. Parent support (Freq)	−0.10^*^	0.10^*^	1				
4. Teacher support (Freq)	−0.13^**^	−0.01	0.37^***^	1			
5. Classmate support (Freq)	−0.02	−0.05	0.40^***^	0.45^***^	1		
6. Close friend support (Freq)	−0.04	−0.00	0.33^***^	0.43^***^	0.59^***^	1	
7. School support (Freq)	−0.01	0.01	0.37^***^	0.38^***^	0.53^***^	0.48^***^	1

^*^p < 0.05,

^**^p < 0.01,

^***^p < 0.001.

### 3.2 Hierarchical regression analysis

Given the study aims, the analyses focused on examining the independent associations of physical activity and social support with BMI Z-scores using hierarchical regression. Therefore, hierarchical regression analyses were used as the primary multivariable approach.

The results of the hierarchical regression analysis in Table 3 show the association with each variable on BMI Z values. In Model 1, the base model with only control variables explained 1.1% of the total variation in BMI Z values (*R*^2^ = 0.011, *F* = 2.78, *p* < 0.10). Of these, gender showed a significant association with BMI Z-scores (β = 0.16, *p* < 0.05), indicating that boys had higher BMI Z values than girls, whereas age was not a significant predictor.

**Table 3:** Hierarchical regression analysis of factors associated with BMI Z-score (*N* = 518).

Variables	Model 1	Model 2	Model 3	Model 4
Control variables				
Age	−0.06	−0.07	−0.07	−0.07
Gender	0.16^*^	0.31^***^	0.31^***^	0.31^***^
Physical activity				
PARS-3 score		−0.34^***^	−0.34^***^	−0.34^***^
Social support				
Teacher frequency			−0.10^**^	−0.10^*^
Parental frequency				−0.06
*R* ^2^	0.011	0.125	0.139	0.141
Adjusted *R*^2^	0.007	0.119	0.132	0.133
Δ*R*^2^	0.011	0.114^***^	0.014^**^	0.003
*F* for Δ*R*^2^	2.78†	66.85^***^	8.36^**^	1.56

^*^p < 0.05.

^**^p < 0.01.

^***^p < 0.001.

Model specifications:

Model 1: Control variables (age, gender).

Model 2: Model 1 + physical activity (PARS-3 score).

Model 3: Model 2 + Teacher Support.

Model 4: Model 3 +Parent Support.

The addition of the physical activity variable to Model 2 significantly increased the explanatory power of the model to 12.5% (*R*^2^ = 0.125), with an increase in *R*^2^ of 11.4% (Δ*R*^2^ = 0.114, *F* = 66.85, *p* < 0.001). The Physical activity showed a strong negative association with BMI Z-scores (β = −0.34, *p* < 0.001), suggesting that a higher level of physical activity was associated with lower BMI Z values. Meanwhile, the predictive effect of gender was further enhanced (β = 0.31, *p* < 0.001), which may be related to differences in physical activity levels between male and female students.

Model 3 added teacher support to the previous model, which further increased the explanatory power of the model to 13.9% (*R*^2^ = 0.139), with a significant increase in *R*^2^ of 1.4% (Δ*R*^2^ = 0.014, *F* = 8.36, *p* < 0.01). Teacher support showed a significant negative association (β = −0.10, *p* < 0.01), indicating that the more teacher support, the lower the student's BMI Z-score. Compared with physical activity, which showed a substantially larger standardized coefficient (β = −0.34), the effect of teacher support was smaller in magnitude but remained statistically significant. Notably, the association of physical activity remained stable (β = −0.34, *p* < 0.001), indicating that teacher support was independently associated with BMI Z-scores after accounting for physical activity and demographic variables.

The addition of parental support to final model 4 slightly increased the total explanatory power of the model to 14.1% (*R*^2^ = 0.141), but the increase in *R*^2^ was only 0.3% and not significant (Δ*R*^2^ = 0.003, *F* = 1.56, *p* > 0.05). The association of parental support was not significant (β = −0.06, *p* > 0.05), whereas the association of teacher support was diminished but still significant (β = −0.10, *p* < 0.05). Physical activity consistently remained the strongest association (β = −0.34, *p* < 0.001), and the role of gender remained stable across all models (β = 0.31, *p* < 0.001).

Taken together, physical activity showed the strongest association with BMI Z-scores among the examined factors, teacher support showed an independent inverse association with BMI Z-scores, and parental support was not a significantly associated with BMI Z values after controlling for other variables. The final model explained a total of 14.1% of the total variance in BMI Z values, with physical activity contributing the main explanatory power. Based on the final model *R*^2^ value, the corresponding Cohen's *f*^2^ was 0.16, indicating a small-to-medium overall effect size.

Based on the significant associated factors in Table 3 hierarchical regression analysis, the present study further used Restricted Cubic Spline (RCS) analysis to explore the pattern of non-linear relationships between physical activity (PARS-3) and frequency of teacher support on BMI Z-scores and to test whether there were gender differences in these relationships.

**Figure 1:**
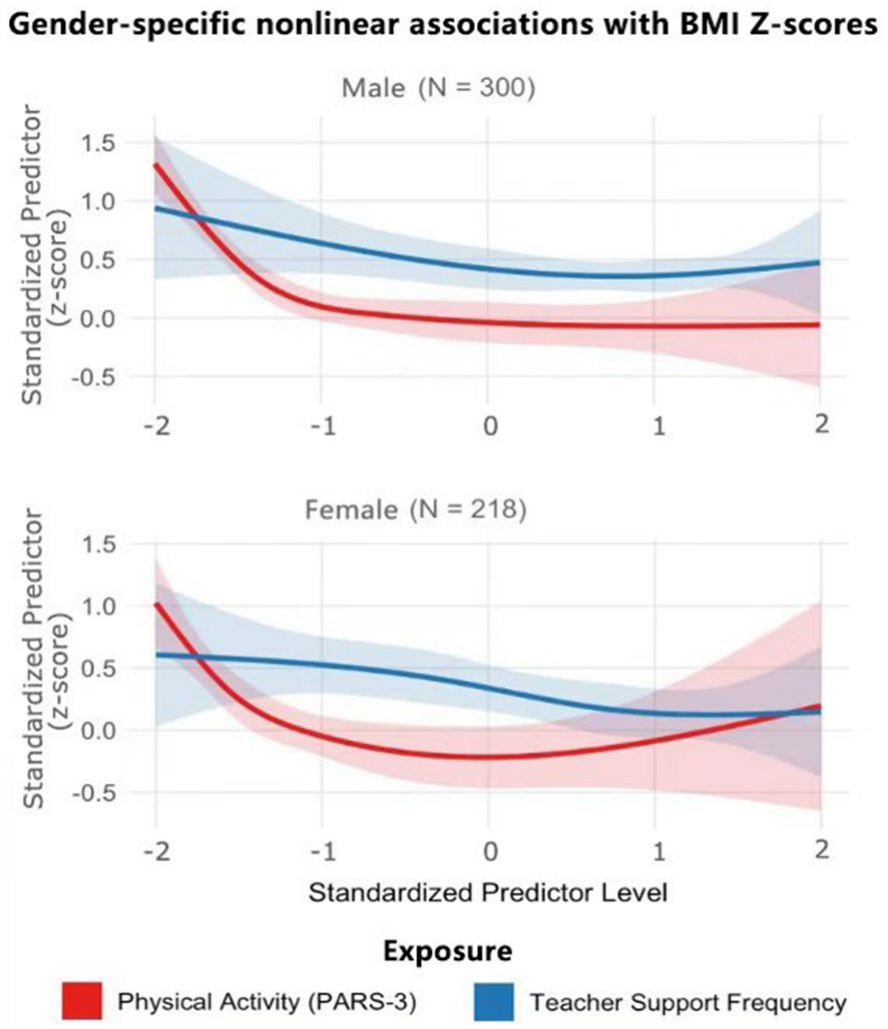
Sex-specific non-linear patterns linking physical activity and teacher support to BMI Z scores. Estimated using restricted cubic spline models. Splines were fitted with three knots placed at the 10th, 50th, and 90th percentiles of the exposure distribution. Shaded areas represent 95% confidence intervals. Estimates at the extreme ends of the physical activity distribution should be interpreted with caution due to lower data density.

As shown in Figure 1, the results of the non-linear test of restricted cubic spline analysis showed that the non-linear relationship between physical activity and BMI Z-score was statistically significant in the total sample (non-linear *P* = 0.032). Gender-stratified analyses showed that the non-linear test was borderline significant in the male group (non-linear *P* = 0.067) and reached a significant level in the female group (non-linear *P* = 0.018). The non-linear test for frequency of teacher support was significant in the female group (non-linear *P* = 0.041) and not significant in the male group (non-linear *P* = 0.234). Based on this statistical evidence, the pattern of non-linear effects for each variable is described separately below.

### 3.3 Non-linear effect of physical activity on BMI Z-score

RCS analyses revealed a clear pattern of non-linear relationship between physical activity and BMI Z-score and showed different characteristics in males and females. In the male population (*N* = 300), physical activity showed a strong dose-response relationship: as the level of physical activity increased from a low level (PARS-3 score ≤ 20) to a moderate level (PARS-3 score 40–60), the BMI Z-score decreased from approximately 1.2 to close to 0.1, a decrease of 1.1 standard deviation units; when physical activity reached a medium high level (PARS-3 score ≥60), the decreasing trend in BMI Z-score gradually leveled off, suggesting a diminishing marginal effect. The linear trend test showed significance (*p* < 0.001), consistent with the strong predictive effect of physical activity in hierarchical regression analysis (β = −0.34, *p* < 0.001).

In contrast, the association between physical activity and BMI Z-score in the female group (*N* = 218) exhibited a U-shaped pattern. At lower levels of physical activity (PARS-3 scores ≤ 25), BMI Z-scores were relatively high (approximately 0.8). As physical activity increased to moderate levels (PARS-3 scores 45–55), BMI Z-scores declined to a nadir of approximately −0.1. However, at higher levels of physical activity (PARS-3 scores ≥70), BMI Z-scores showed a modest rebound to around 0.2. The quadratic term for this U-shaped association reached borderline statistical significance (P = 0.052). It should be noted that the rebound observed at higher physical activity levels occurred at the upper end of the exposure distribution, where fewer observations were available, and should therefore be interpreted with caution.

### 3.4 The model of the protective effects of teacher support

The effect of teacher support frequency on BMI Z-scores also showed clear gender differences. Among boys, teacher support demonstrated a modest protective effect: as support frequency increased from lower to higher levels, BMI Z-scores declined by approximately 0.4 standard deviation units, with a significant linear trend (*P* = 0.028).

This protective association was more pronounced and consistent among girls. BMI Z-scores decreased steadily from approximately 0.5 at lower levels of teacher support to around 0.1 at higher levels, representing a reduction of about 0.4 standard deviation units, with a highly significant linear trend (*P* = 0.003). These findings indicate a stronger and more stable association between teacher support and BMI Z-scores in girls compared with boys.

### 3.5 The difference in the relative importance of the predictors by gender

Gender differences in predictor effects could be quantified by comparing the slopes and magnitude of change in the RCS curves. Among males, the maximum effect size (magnitude of change in BMI Z-score from low to high levels) was 1.1 standard deviation units for physical activity and 0.4 standard deviation units for teacher support, with the effect of physical activity being approximately 2.8 times greater than that of teacher support. Among females, the maximum effect sizes were 0.9 standard deviation units for physical activity and 0.4 standard deviation units for teacher support, for an effect ratio of 2.3 times, indicating that the difference between the effects of the two factors was relatively small among females.

This quantitative result is consistent with the finding of physical activity as the strongest associated factor in the regression analyses (Table 3) (β = −0.34, *p* < 0.001), and reveals the specific extent of the gender difference: males responded more sensitively to physical activity, while the relative importance of teacher support increased among females. Teacher support had similar effect sizes in both gender groups, but the relative attenuation of the physical activity effect in females made teacher support more prominent in female weight management.

## 4 Discussion

This study systematically explored the predictive role of multiple sources of social support (parents, teachers, classmates, close friends, and school) and physical activity on the BMI Z-score of Chinese junior high school students. This study provides an integrated examination of how physical activity and multiple sources of social support relate to BMI Z-scores among Chinese junior high school students. The findings indicate that behavioral factors and school-based social contexts jointly contribute to weight-related outcomes, with clear gender-specific patterns observed across these associations.

### 4.1 Core predictive role of physical activity: consistent with trends in international evidence

Physical activity demonstrated the strongest standardized association with BMI Z-scores in the present models (β = −0.34, *p* < 0.001), accounting for the largest proportion of the explained variance. From an effect size perspective, the overall model yielded a small-to-medium effect (Cohen's *f*^2^ = 0.16), which is considered meaningful given the multifactorial nature of adolescent BMI and the influence of multiple biological, behavioral, and social determinants. Although the independent association of teacher support (β = −0.10) was smaller in magnitude compared with physical activity, it represents a modifiable school-context factor that may exert indirect influences on weight-related outcomes through behavioral and psychosocial pathways. Consistent with our findings, a large-sample study conducted in the Chengdu–Chongqing economic region of China reported that moderate- to high-intensity physical activity significantly reduced obesity prevalence among adolescents (Liu et al., 2024). Similarly, a study of junior high school students in Shanghai found that moderate-intensity physical activity was inversely associated with BMI, whereas high-intensity physical activity showed a positive association (Bai et al., 2024). Longitudinal evidence from the United Kingdom indicates that MVPA among children aged 6–11 years declined by approximately 2.2 min. per day per year, with disparities in activity levels between BMI categories widening over time (Jago et al., 2020). At the global level, WHO data suggest that the prevalence of overweight among adolescents increased from 8% in 1990 to 20% in 2022, with particularly pronounced trends observed in the Asian region (World Health Organization, 2024). Within this global context, the comparatively strong association between physical activity and BMI Z-scores observed in Chinese junior high school students underscores the central role of physical activity in adolescent weight regulation and provides localized empirical support for the development of targeted intervention strategies. This finding also establishes an important foundation for subsequent analyses of gender-specific patterns and the potential role of social support mechanisms.

### 4.2 The role of physical activity and gender-specific patterns

While physical activity showed the strongest overall association with BMI Z-scores, the restricted cubic spline analyses revealed that this association was not uniform across genders. Instead, distinct dose–response patterns were observed for boys and girls, suggesting that the physical activity–BMI relationship may operate differently during early adolescence. Notably, the RCS analyses suggested gender-specific patterns, with a largely linear dose–response relationship observed in males and a non-linear pattern observed in females. Although non-linear associations between physical activity and health-related outcomes are less commonly reported in adolescents, similar U-shaped patterns have been documented in related contexts, such as associations between BMI and physical fitness indicators among Chinese university students and between physical activity and academic performance among Korean female adolescents (Wu H. et al., 2024; Jee, 2025).

The U-shaped association observed in girls may reflect complex psychophysiological and psychosocial mechanisms. Previous research suggests that adolescent girls may be particularly sensitive to body image concerns and exercise-related psychosocial pressures, which could influence weight-related outcomes at higher levels of physical activity (Voelker et al., 2015). However, given the cross-sectional design and the lower data density at the upper end of the physical activity distribution, this pattern should be interpreted with caution. In particular, the rebound in BMI Z-scores at very high levels of physical activity may represent an early manifestation of non-linear associations during early adolescence rather than a stable dose–response threshold. From a practical perspective, these findings suggest the presence of potentially gender-specific patterns when examining physical activity–BMI associations, rather than prescribing uniform activity thresholds.

### 4.3 The unique role of teacher support: the discovery of differences in cultural contexts

The present study highlights the prominence of teacher support among multiple sources of social support. Not only was the frequency of teacher support significantly and inversely associated with BMI Z-scores (*r* = −0.13, *p* < 0.01), but it also remained an independent correlate after controlling for physical activity and other social support sources (β = −0.10, *p* < 0.01), explaining an additional 1.4% of the variance. This finding contrasts with much of the Western literature, which often emphasizes parental or peer support as primary social determinants of adolescent health behaviors. Such differences may be partly attributable to variations in the relative roles of parents, peers, and teachers across cultural and educational contexts.

In Western contexts, parental support and support from classmates or close friends are frequently identified as key influences on adolescent physical activity and related health behaviors. For example, studies conducted in Brazil have reported that friend encouragement is a significant predictor of vigorous physical activity among adolescent girls (de Camargo et al., 2023). In contrast, the present study found that peer and friend support were not significantly associated with BMI Z-scores, a pattern that may reflect the distinctive structure of the Chinese educational environment.

The decision to focus on parental and teacher support in the final regression models was guided by both theoretical considerations derived from the social–ecological framework and the specific developmental context of Chinese junior high school students. Within this framework, parents and teachers represent primary adult authority figures who structure adolescents' daily routines, educational priorities, and opportunities for physical activity, particularly during the compulsory education stage in China. Although peer and friend support have been consistently linked to adolescents' physical activity behaviors in Western studies (Laird et al., 2016; Lisboa et al., 2021), existing evidence suggests that peer-related effects often operate indirectly through mediators such as self-efficacy, enjoyment, or social norms rather than exerting a direct influence on weight-related outcomes such as BMI.

Recent Chinese research has further reported beneficial associations between teacher academic support and adolescent behavioral adjustment, potentially through mechanisms such as enhanced self-efficacy and a stronger sense of school belonging (Fang et al., 2025). Within the Confucian cultural tradition, teacher–student relationships carry connotations of authority and trust, which may increase adolescents' receptivity to teachers' guidance (Wang et al., 2024). This cultural context may help explain why teacher support showed an independent association with BMI Z-scores in the present study. Non-etheless, the non-inclusion of peer and classmate support in the final models may have led to an underestimation of indirect or synergistic effects, particularly along physical activity pathways. This limitation may be especially relevant in gender-specific analyses, and future studies using mediation or structural equation modeling approaches are warranted to further elucidate the complex and potentially gender-specific mechanisms through which multiple sources of social support jointly influence physical activity and weight-related outcomes.

### 4.4 Differences in the effects of parental and peer support in the Chinese context

Parents' support, classmates' support, and close friend support did not show significant predictive effects on BMI Z-scores in the present study. This finding should be interpreted within the specific sociocultural and educational context of Chinese junior high school students, rather than as evidence that these sources of support are universally unimportant. It is worth noting that findings regarding the effects of parental and peer support on adolescent physical activity and weight-related outcomes have been inconsistent across studies. On the one hand, a large-scale study from Wuhan University reported that parental support had a significant positive effect on moderate-to-vigorous physical activity through the mediation of family physical activity resource availability and adolescents' autonomous motivation (Zeng et al., 2022). Similarly, a study based on 2,341 Chinese adolescents found that social support was significantly and positively associated with physical activity (Ren et al., 2020). One possible explanation for the non-significant effect of parental support in the present study is the “academic-first” orientation commonly observed among Chinese parents during the junior high school stage. Under conditions of high academic pressure and competitive examination systems, parental involvement may be more strongly directed toward academic supervision than toward the promotion of physical activity, potentially weakening its observable association with BMI-related outcomes.

On the other hand, previous studies have suggested that the effects of parental and peer support may be conditional or indirect. For example, peer support has been shown to influence adolescent physical activity primarily through mediators such as self-efficacy and exercise enjoyment, rather than exerting a direct effect (Chen et al., 2017; Zou et al., 2023). Longitudinal evidence from Poland further indicates that parental social control and support may be effective only among children with low to moderate BMI Z-scores, with diminished effects observed among those with higher BMI Z-scores (Liszewska et al., 2018).

The present study employed the CASSS scale, which distinguishes social support across multiple sources (parents, teachers, classmates, close friends, and school) and dimensions (frequency and importance). As the current analyses focused on frequency-based indicators of received support, indirect or qualitative aspects of parental and peer support may not have been fully captured. This contrasts with studies using global social support measures, suggesting that differences in measurement approaches may contribute to discrepancies in observed associations (Kiechle et al., 2015).

### 4.5 Limitations

Several limitations should be acknowledged. First, due to the cross-sectional design, causal inferences cannot be made. In particular, the observed inverse association between teacher support and BMI Z-scores may reflect reverse causality, whereby students with lower BMI are more likely to receive or perceive greater teacher support. Therefore, the protective effect of teacher support observed in this study should be interpreted as associational rather than causal.

Second, although hierarchical regression was applied, the assumptions underlying linear regression may not be fully satisfied in observational school-based data. As with similar studies, potential deviations from assumptions such as linearity, normality, and homoscedasticity cannot be completely ruled out and may have influenced the magnitude of the estimated associations. In addition, although BMI Z-scores showed moderate variability, descriptive statistics were reported using mean and standard deviation, which is appropriate for regression-based analyses. As the primary analyses relied on regression models rather than group comparisons, strict normality of individual variables was not a prerequisite. Moreover, the final model explained a modest proportion of the variance in BMI Z-scores, indicating that additional unmeasured factors may contribute to weight-related outcomes in this population.

In addition, although students were sampled from multiple schools, school-level clustering was not explicitly modeled, and potential contextual differences between schools cannot be entirely excluded. Physical activity was assessed using a self-reported questionnaire, which may be subject to recall and social desirability bias. Moreover, the PARS-3 captures overall physical activity intensity, duration, and frequency, but does not distinguish between specific activity domains such as school-based activity, transportation, or leisure-time physical activity. Therefore, the present findings reflect associations with total physical activity rather than domain-specific patterns. Future longitudinal studies using objective and domain-specific measures are needed to clarify the directionality and robustness of these associations. Furthermore, although BMI Z-scores are widely used for assessing weight status in pediatric populations, BMI remains a proxy indicator of adiposity and may not fully capture body composition changes during adolescence, a period characterized by rapid growth and pubertal development.

## 5 Conclusions

This study provides evidence that teacher support is a salient school-based correlate of BMI Z-scores among Chinese junior high school students, highlighting a pattern that differs from much of the Western literature, which more often emphasizes parental and peer influences. Physical activity remained the strongest correlate of weight status; however, its associations with BMI Z-scores differed by gender, suggesting that activity–weight relationships may operate differently for boys and girls during early adolescence. The relative prominence of teacher support over other sources of social support reflects the distinctive characteristics of the Chinese educational context and underscores the importance of the school setting in adolescent health promotion. From a policy perspective, these findings suggest that school-based obesity prevention efforts in China may benefit from strengthening teachers' supportive roles, particularly within physical education settings. Practical approaches may include targeted training for PE teachers to provide positive feedback, emotional encouragement, and inclusive participation opportunities, with particular attention to engaging girls, who appeared to show greater responsiveness to teacher support in this study.

## Data Availability

The datasets used and/or analyzed during the current study are available from the corresponding author on reasonable request.
